# A Linear Mixed Model Spline Framework for Analysing Time Course ‘Omics’ Data

**DOI:** 10.1371/journal.pone.0134540

**Published:** 2015-08-27

**Authors:** Jasmin Straube, Alain-Dominique Gorse, PROOF Centre of Excellence Team, Bevan Emma Huang, Kim-Anh Lê Cao

**Affiliations:** 1 QFAB Bioinformatics, Institute for Molecular Bioscience, University of Queensland, Brisbane, QLD, Australia; 2 Prevention of Organ Failure (PROOF) Centre of Excellence, University of British Columbia and Providence Health Care, Vancouver, BC, Canada; 3 CSIRO Digital Productivity Flagship, Brisbane, QLD, Australia; 4 The University of Queensland Diamantina Institute, Translational Research Institute, Brisbane, QLD, Australia; Oregon State University, UNITED STATES

## Abstract

Time course ‘omics’ experiments are becoming increasingly important to study system-wide dynamic regulation. Despite their high information content, analysis remains challenging. ‘Omics’ technologies capture quantitative measurements on tens of thousands of molecules. Therefore, in a time course ‘omics’ experiment molecules are measured for multiple subjects over multiple time points. This results in a large, high-dimensional dataset, which requires computationally efficient approaches for statistical analysis. Moreover, methods need to be able to handle missing values and various levels of noise. We present a novel, robust and powerful framework to analyze time course ‘omics’ data that consists of three stages: quality assessment and filtering, profile modelling, and analysis. The first step consists of removing molecules for which expression or abundance is highly variable over time. The second step models each molecular expression profile in a linear mixed model framework which takes into account subject-specific variability. The best model is selected through a serial model selection approach and results in dimension reduction of the time course data. The final step includes two types of analysis of the modelled trajectories, namely, clustering analysis to identify groups of correlated profiles over time, and differential expression analysis to identify profiles which differ over time and/or between treatment groups. Through simulation studies we demonstrate the high sensitivity and specificity of our approach for differential expression analysis. We then illustrate how our framework can bring novel insights on two time course ‘omics’ studies in breast cancer and kidney rejection. The methods are publicly available, implemented in the R CRAN package lmms.

## Introduction

Over the past decade, the use of ‘omics’ to take a snapshot of molecular behaviour has become ubiquitous. It has recently become possible to examine a series of such snapshots by measuring an ‘ome’ over time. This provides a powerful tool to study stressor-induced molecular behaviour [1], developmental processes (e.g., ageing; [2]) and cyclic mechanisms (e.g., cell cycle; [3]).

Robust and powerful analysis tools are critical for capitalizing on the wealth of data to answer key questions about system response and function. In addition to addressing the high-dimensionality of the data, such tools must account for a high number of missing values, and also variability within and between studied subjects. Many methods are limited by scale, and are unable to handle either a large number of time points, a varying number of time points per subject [4] or a very large number of molecules [5]. Hence there is an urgent need for filtering and modelling these time course data, not only to decrease the number of profiles analyzed, but also to collapse subject-specific profiles to a summary thereof.

The benefit of decreasing the number of profiles analyzed via filtering is evident when considering the scale of typical time course ‘omics’ experiments. Tens of thousands of molecules can be measured at different time points, requiring multiple hypothesis tests to determine differential expression. While the false positive rate can be controlled using multiple testing corrections (e.g., FDR; [6]), these are frequently accompanied by an increase in the false negative rate. Hence identifying and removing non-informative molecules prior to testing can help to increase statistical power. This drives a need for accurate approaches to remove a large number of non-informative profiles. Indeed, estimates are that only 30–40% of the genes are expressed at array-detectable levels [7], increasing up to 60–70% for newer technologies like RNA sequencing [8]. Furthermore, modelling can provide considerable benefits by summarizing the remaining, informative profiles. Our aim in this study is to model the systematic process from which expression levels derive, as a smooth function over time, so that observed measurements can then be seen as a noisy realization of this function.

A popular modelling approach for time course data is smoothing splines, which use a piecewise polynomial function with a penalty term [9]. The two main drawbacks are the arbitrary selection of the penalty and the computational burden, both of which have received extensive attention. For example, [10] reparametrized smoothing splines in a linear mixed model spline framework to address the arbitrary choice of penalty. However, the smoothing splines models developed in this framework are still computationally challenging to fit with an increasing number of time points [11, 12]. The standard smoothing splines approach faces similar challenges, which can in part be mitigated using spline regression. There the computation-limiting factor is the number of polynomial pieces rather than the number of time points. Since splines can be calculated using linear mixed models, a wide range of methods have been proposed to improve computational efficiency of such models [13], [14]. More recently, [15] presented a tradeoff between spline regression and the linear mixed model spline framework by combining low-rank smoothers adapted from [16] with the penalty approach of [13]. The hybrid approach results in a truncated spline basis which improves computational efficiency and relaxes the importance of initial parameters choices.

After the filtering and modelling steps, the resulting summarized profiles can be clustered to gain biological insight from their similarities. Indeed, clusters of correlated activity patterns may predict putative functions for molecules and reveal stage- and tissue-specific regulators [2]. To that end, several spline-based clustering methods have been proposed in the literature [17, 18]. However, common limitations include additional assumptions on the distribution of the data, computational cost and dependency of the resulting clusters on the initial parameters. To our knowledge, no approach currently incorporates subject-specific random effects in a spline model in order to accurately model subject-specific variation before clustering.

Hypothesis testing can also be performed within the mixed effect model framework to gain biological insight from differences between groups and across time. Several methods have been proposed which can all handle missing data and different numbers of replicates per time point, but are often limited when only a few time points are observed, as is typically the case for costly high-throughput experiments. Approaches such as linear models for microarray data (LIMMA; [19]) test contrasts of interest in a spline framework using an empirical Bayes approach [20], but do not account for subject-specific variation in the model. Extraction and analysis of Differential Gene Expression (EDGE; [21]) does model subject-specific effects as scalar shifts from the mean population response but lacks flexibility and has been reported to not adequately model data in simulated scenarios [22]. A more flexible approach is Smoothing Splines Mixed Effects (SME; [22]), which models subject-specific effects as full curves, but with the risk of over-smoothing profiles in some cases.

**Fig 1:**
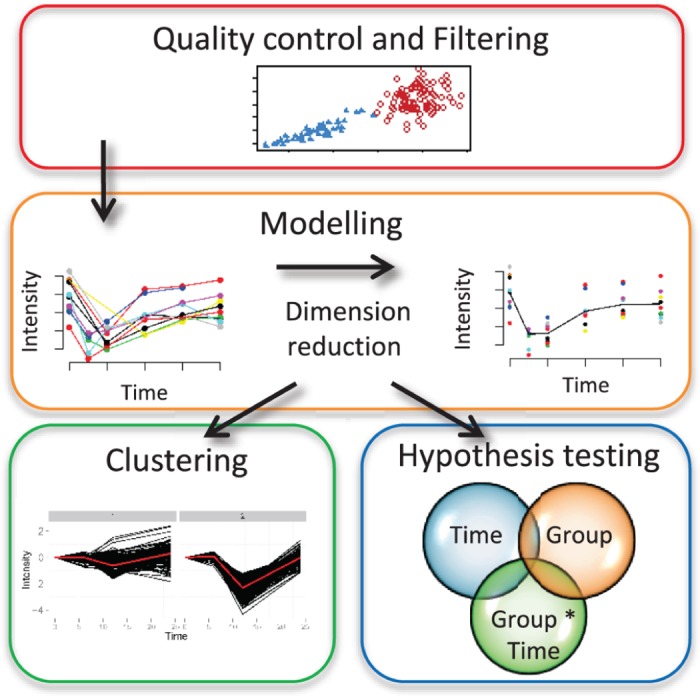
Overview of the analysis framework. The proposed framework consists of three stages: quality control and filtering; serial modelling of profiles; and analysis with clustering to identify similarities between profiles or with hypothesis testing to identify differences over time, between groups, and/or in group and time interactions.

In this paper we propose a novel framework for time course ‘omics’ studies which is summarized in Fig 1). First, we extend a quality assessment and filtering approach to time course data to identify and remove non-informative molecular profiles. Second, we propose a serial modelling approach which avoids both under- and over- smoothing by allowing the data to drive the complexity of the curve in order to fit the appropriate model. These modelled and summarized profiles can then be analyzed for clustering and differential expression analyses. We illustrate the use of our framework in simulation and real time course ‘omics’ case studies.

## Material and Methods

### Material

We first applied the filtering and modelling stages of our framework to two publicly available transcriptomics datasets, which are briefly described below. The main analyses and biological interpretations were then performed on two proteomics datasets from breast cancer and kidney rejection studies.

#### *S. paradoxus* evolution data (GSE36253)

The evolutionary principles of modular gene regulation in yeast were investigated by [23]. They tracked growth on glucose in real-time by measuring the growth rate, glucose, and ethanol levels. Expression of 5,503 genes was measured at six physiologically comparable time points. Samples were hybridized to microarrays with the reference chosen to be the same physiological phase in all cases. In this study we selected a single species (*S. paradoxus*) with two to four biological replicates per time point.

#### *M. musculus* chemoimmunotherapy data (GSE27440)

The anti-tumour efficiency of a chemotherapeutic drug on bone marrow in mice was investigated by [24]. Expression of 13,443 genes was measured pre-treatment, 1, 2 and 5 days after chemotherapy of tumour-bearing mice. At each time point five biological and two technical replicates were assayed.

#### iTraq breast cancer data (PRD000178)

Proteomic changes in MCF-7 cells resulting from insulin-like growth factor 1 (IGF-1) stimulation were investigated by [1]. As impairment of the IGF-1 receptor signalling network is involved in tumour growth and chemotherapy resistance, the study of proteins involved in this network may help to understand the underlying mechanisms and to identify potential drug targets. iTraq Liquid Chromatography followed by a two-dimensional Mass Spectrometry scan (LC-MS/MS) was used to quantify proteins at 0 h (no IGF-1), 6, 12 and 24 h after IGF-1 stimulation. This procedure was repeated in three separate cultures. In total 899 proteins were identified. Sample-wise scaled *log*_2_ fold changes for time points 6, 12 and 24 h relative to baseline (0 h) were reported for 264 proteins with minimum two measured replicates. We applied our full data-driven modelling approach to this dataset, finishing with cluster analysis to explore patterns of protein response to IGF-1 stimulation.

#### iTraq kidney rejection data

The PROOF Centre of Excellence performed a longitudinal study to identify diagnostic biomarkers in blood plasma to predict acute renal allograft rejection [25]. The iTraq kidney rejection dataset is a subsample thereof which includes 10 Acute Rejection patients (AR) and 20 Non-Rejection patients (NR). In this discovery study, iTraq MALDI-TOF MS/MS technology was used to quantify plasma protein relative concentrations in blood samples tracked prior to (0 weeks) and post transplant at 0.5, 1, 2, 3, and 4 weeks. In total, 140 proteins were quantified from blood samples. We applied our full data-driven modelling approach to this dataset, finishing with differential expression analysis to identify proteins whose profiles differed between the two groups.

#### Simulated data

For each of six different scenarios varying noise levels and fold changes, we simulated 100 datasets, each consisting of 140 profiles, 50 of which were differentially expressed. For each dataset, we applied our differential expression approach and LIMMA [19], and compared their sensitivity and specificity to differential expression over time, between groups and in group*time interactions. A detailed description of the simulation procedure can be found in the Supporting Information files, with examples of simulated profiles (Figure A in S1 File).

## Methods

### Quality control and filtering

Filtering on the overall standard deviation of molecule expression is a common approach in static gene expression experiments to remove non-informative molecules prior to analysis [26]. The justification is that low standard deviations indicate little molecular activity, and so molecules which vary more are of more interest. In time course experiments however, molecules can vary both over time and between subjects. Therefore, an increase in the overall standard deviation does not necessarily indicate interesting molecular behaviour and the additional time dimension of the data needs to be accounted for.

Rather than the overall standard deviation, defined below as *s*_*M*_, we considered two filter ratios based on the standard deviations across time and subjects. These estimates can be used to identify low quality and/or non-informative profiles. Let *T* be the number of time points and *n* the total number of subjects. For each molecule, we denote by *y*_*i*_(*t*) the expression for subject *i* at time *t*, with *i* = 1, …, *n* and by *s*_*T*_ the average of standard deviations (SD) computed per time point with

$$\begin{array}{c} s_{T}=\frac{1}{T}\sum_{t=1}^{T}\sqrt{\frac{1}{n-1}\sum_{i=1}^{n}{\left(y_{i}\left(t\right)-\mu_{t}\right)}^{2}},\;\text{where}\;\mu_{t}=\frac{1}{n}\sum_{i=1}^{n}y_{i}\left(t\right). \end{array}$$

Similarly, *s*_*I*_ is the average of SDs computed per subject, with

$$\begin{array}{c} s_{I}=\frac{1}{n}\sum_{i=1}^{n}\sqrt{\frac{1}{T-1}\sum_{t=1}^{T}{\left(y_{i}\left(t\right)-\mu_{i}\right)}^{2}},\;\text{where}\;\mu_{i}=\frac{1}{T}\sum_{t=1}^{T}y_{i}\left(t\right), \end{array}$$

and *s*_*M*_ is the SD for each molecule, over all subjects and time points:

$$\begin{array}{c} \begin{array}{c} s_{M}=\sqrt{\frac{1}{Tn-1}\sum_{t=1}^{T}\sum_{i=1}^{n}{\left(y_{i}\left(t\right)-\mu_{M}\right)}^{2}}, \end{array}\;\text{where}\;\mu_{M}=\frac{1}{Tn}\sum_{t=1}^{T}\sum_{i=1}^{n}y_{i}\left(t\right). \end{array}$$

Missing values were excluded from the relevant sums. We then define the filter ratios *R*_*T*_ and *R*_*I*_ as

$$\begin{array}{c} R_{T}=\frac{s_{T}}{s_{M}}\;\text{and}\;R_{I}=1-\frac{s_{I}}{s_{M}}. \end{array}$$

**Fig 2:**
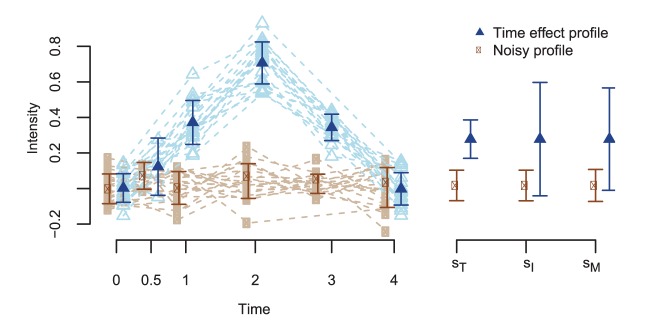
Examples of ‘noisy’ and differentially expressed profiles. Profiles changing over time (blue) have a mean of the standard deviations per time point (*s*_*T*_) smaller than the mean of the standard deviations per molecule (*s*_*M*_), while these means have similar values for noisy molecules (brown). In both cases the mean of the standard deviations per subject (*s*_*I*_) is similar to *s*_*M*_.

Our filter ratios are motivated by the expectation that the SD values for profiles consisting purely of noise are different compared to those with a true signal over time. Fig 2 illustrates some example profiles to motivate the use of one of the ratios, *R*_*T*_, for quality control. The first type of profile consists purely of noise, resulting in *s*_*T*_ ≈ *s*_*M*_ and therefore *R*_*T*_ ≈ 1. The second type of profile has a true signal over time, resulting in *s*_*M*_ greater than *s*_*T*_ and *R*_*T*_ < 1. Hence, *R*_*T*_ provides one means of discriminating between non-informative and informative profiles. We generally expect subject-specific profiles to be close to the mean molecule profile, resulting in *R*_*I*_ ≈ 0, as would also be true for noisy profiles over time. Therefore, on its own, *R*_*I*_ is only a good discriminator of unambiguously flat profiles, for which *s*_*I*_ may often be smaller than *s*_*M*_, resulting in *R*_*I*_ > 0. Nevertheless, the combination of both *R*_*T*_ and *R*_*I*_ can provide additional insights into the variance structure of the molecules and can guide the user to make more informed choices about filter ratio thresholds as illustrated in our case studies.

During our filtering stage, we first removed molecules with more than 50% missing data and applied model-based clustering (R package mclust [27]) on the filter ratios *R*_*T*_ and *R*_*I*_ by specifying two clusters. Based on the rationale described above, we expect the cluster of profiles with low *R*_*T*_ and *R*_*I*_ to be informative and propose to discard profiles in the cluster with high *R*_*T*_ and *R*_*I*_. In the specific case where a time course study includes the comparison of multiple conditions or treatments, it is important to avoid filtering profiles which may be non-informative within a condition but are differentially expressed between conditions. Therefore, we propose to apply the filtering approach to each condition separately, with the additional requirement that profiles must be found non-informative in all conditions in order to be removed.

### Modelling

In high-throughput experiments, thousands of molecule profiles need to be modelled in an efficient manner. Biological variability both between and within subjects must be accounted for, and experimental procedures typically result in different numbers of replicated measurements per molecule and time point. The combination of all of these factors requires a flexible, robust model-fitting procedure which can easily accommodate different sources of variation.

**Model fit with Linear Mixed Model Splines (LMMS)**: For each molecule, we determine an appropriate model via a serial model fitting approach. This avoids under- or over-fitting by allowing the data structure to drive the model complexity, rather than relying on *a priori* assumptions such as in [19], [21]. We make comparisons between successive models using a goodness of fit test, retaining a more complex model only if it fits the data better than a simpler model. The goodness of fit is assessed with the log likelihood ratio test as implemented in the anova function of the nlme package. The four models considered in this process are described below, listed in order of increasing complexity.

The first model assumes the response is a straight line and is not affected by subject variation. For each molecule, we denote by *y*_*ij*_(*t*_*ij*_) its expression for subject (or biological replicate) *i* at time *t*_*ij*_, where *i* = 1, 2, …, *n*, *j* = 1, 2, …, *m*_*i*_, *n* is the sample size and *m*_*i*_ is the number of observations for subject *i*. We fit a simple linear regression of expression *y*_*ij*_(*t*_*ij*_) on time *t*_*ij*_, where the intercept *β*_0_ and slope *β*_1_ are estimated via ordinary least squares:

$$\begin{array}{c} y_{ij}\left(t_{ij}\right)=\beta_{0}+\beta_{1}t_{ij}+\epsilon_{ij},\;\text{where}\;\epsilon_{ij}\;∼\;N\left(0,\sigma_{\epsilon }^{2}\right). \end{array}$$ (1)

As nonlinear response patterns are commonly encountered in time course biological data [28], our second model replaces the straight line in Eq 1 with a curve that is modelled using a spline truncated line basis as proposed by [15]:

$$\begin{array}{c} y_{ij}\left(t_{ij}\right)=f\left(t_{ij}\right)+\epsilon_{ij},\;\text{where}\;\epsilon_{ij}∼N\left(0,\sigma_{\epsilon }^{2}\right). \end{array}$$ (2)

In Eq 2 *f* represents a penalized spline which depends on a set of knot positions *κ*_1_, …, *κ*_*K*_ in the range of {*t*_*ij*_}, some unknown coefficients *u*_*k*_ to be estimated, an intercept *β*_0_ and a slope *β*_1_. That is,

$$\begin{array}{c} f\left(t_{ij}\right)=\beta_{0}+\beta_{1}t_{ij}+\sum_{k=1}^{K}u_{k}{\left(t_{ij}-\kappa_{k}\right)}_{+},\\ \text{with}\;\;{\left(t_{ij}-\kappa_{k}\right)}_{+}=\{\begin{array}{cc} t_{ij}-\kappa_{k} & \text{if}\;t_{ij}-\kappa_{k}>0,\\ 0 & \text{otherwise.} \end{array} \end{array}$$ (3)

Since a spline is a composition of curve segments and the knots define the break points of the curve segments, the choice of the number of knots *K* and their positions influences the shape of the curve. As proposed by [29], we estimate the number of knots based on the number of measured time points *T* as $K=max(5,min(\lfloor \frac{T}{4}\rfloor ,40))$, setting knots *κ*_1_…*κ*_*K*_ at quantiles of the time interval of interest.

In order to account for subject variation, our third model Eq (4) adds a subject-specific random effect *U*_*i*_ to the mean response *f*(*t*_*ij*_). Assuming *f*(*t*_*ij*_) to be a fixed (yet unknown) population curve, *U*_*i*_ is treated as a random realization from an underlying Gaussian distribution independent from the previously defined random error term *ϵ*_*ij*_. Hence, the subject-specific curves are expected to be parallel to the mean curve as we assume the subject-specific random effects to be constant over time:

$$\begin{array}{c} y_{ij}\left(t_{ij}\right)=f\left(t_{ij}\right)+U_{i}+\epsilon_{ij},\;\text{where}\;U_{i}∼N\left(0,\sigma_{U}^{2}\right). \end{array}$$ (4)

A simple extension to this model is to assume that the subject-specific deviations are straight lines. Our fourth model therefore fits subject-specific random intercepts *a*_*i*0_ and slopes *a*_*i*1_:

$$\begin{array}{c} \begin{array}{c} y_{ij}\left(t_{ij}\right)=f\left(t_{ij}\right)+a_{i0}+a_{i1}t_{ij}+\epsilon_{ij},\;\\ \text{with}\;\epsilon_{ij}\;∼N\left(0,\sigma_{\epsilon }^{2}\right)\;\text{and}\;{\left(a_{i0},a_{i1}\right)}^{T}∼N\left(0,\Sigma \right). \end{array} \end{array}$$ (5)

We assume independence between the random intercept and slope, and therefore the covariance matrix for the random effects Σ is diagonal.

**Derivative information for Linear Mixed Model Splines (DLMMS)**: The derivative of expression profiles contains valuable information about the rate of change of expression over time [9, 30]. We consider the derivative of the mean population curve *f*(*t*) from Eq 3. Note that for profiles modelled using only Eq 1 the derivative is constant and is equal to the estimate of the slope. Otherwise, the derivative at any time point *t* in the relevant time interval is:

$$\begin{array}{c} f^{\prime }\left(t\right)={\hat{\beta }}_{1}+\sum_{k=1}^{K}{\hat{u}}_{k}I\left(t,\kappa_{k}\right)\;\text{with}\;I=\{\begin{array}{cc} 1 & \text{if}\;t-\kappa_{k}\geq 0,\\ 0 & \text{otherwise,} \end{array} \end{array}$$

where ${\hat{\beta }}_{1}$ and ${\hat{u}}_{k}$ denote the estimates of the intercept and spline coefficients. The derivatives of the LMMS profiles can then be used instead of the modelled profiles to gain new insights in the downstream cluster analysis.

### Clustering

Clustering of time profiles allows insight into which molecules share similar patterns of response, which may in turn indicate a shared biological basis. Similarities between trajectories may be seen not only in terms of shape and magnitude, but also rates of change, or speed. However, detecting these similarities can be challenging due to noise and missing values in subject-specific measurements. Hence, the choice of modelling approach often has critical impact on the ability to identify clusters of biologically similar molecules.

**Fig 3:**
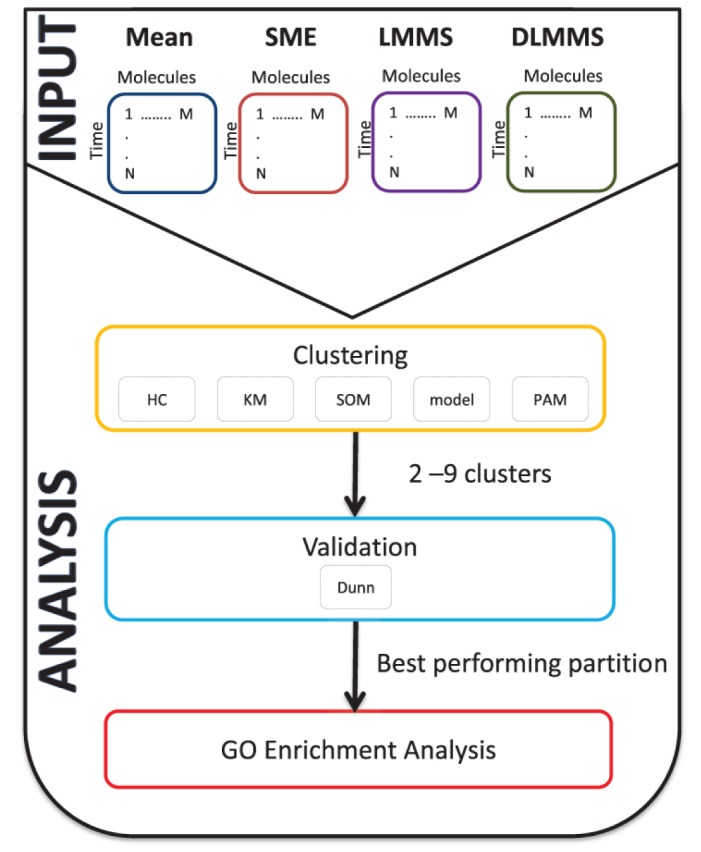
Workflow for the profile cluster analysis. Trajectories derived from Linear Mixed Model Spline (LMMS) and Derivative Linear Mixed Model Spline (DLMMS) were compared to trajectories derived either from the mean or Smoothing Splines Mixed Effects (SME) models. Five clustering algorithms—hierarchical clustering (HC), kmeans (KM), Self-Organizing Maps (SOM), model-based (model) and Partitioning Around Medoids (PAM) were then applied on modelled trajectories using a range of two to nine clusters. The performance of each algorithm was assessed using the Dunn index. Gene Ontology (GO) term enrichment analysis was performed on each of the obtained clusters.

We compared our modelling approaches LMMS and DLMMS to two single-step models using the workflow shown in Fig 3. As a basic comparison, we first calculated the mean at each time point for each molecule as it is arguably the most common way of reducing subject dimension. As a more sophisticated alternative, we applied the R package implementation of the recently proposed modelling approach Smoothing Splines Mixed Effects (SME) [22], which uses a single model that treats each subject-specific trajectory as a smooth function of time.

For clustering, we compared the performance of five algorithms using the Dunn index [31] from the clValid R package [32]. The Dunn index is the ratio of the smallest inter-cluster distance to the largest intra-cluster distance. A large index value indicates a good separation of the clusters, and is our criterion of choice to determine both the appropriate number of clusters and the best performing clustering algorithm.

We selected clustering algorithms for comparison based on representatives of different classes of standard techniques: a model-based algorithm (mclust; [27]), hierarchical clustering, k-means, partitioning around medoids (cluster; [33]), and Self-Organizing Maps (kohonen; [34]). The last four algorithms utilize a dissimilarity metric to cluster profiles derived from SME, mean and LMMS and the Euclidean distance metric for DLMMS.

A size-based Gene Ontology (GO) term enrichment analysis was then performed to validate the biological relevance of each cluster, using the hypergeometric distribution based on the number of molecules in the domain of interest [35]. We specifically examined the molecules’ spatial link (Cellular Compartment), basal activity (Molecular Function) and involvement in a series of molecular events (Biological Process). All annotations were obtained from the org.Hs.eg.db R package [36].

### Differential expression analysis

While cluster analysis can provide valuable insight into behaviour patterns common to groups (clusters) of molecules, differential expression analysis in a time course experiment can highlight significant responses to perturbations of each molecule. Our LMMS framework enables assessment of the significant differences over time or between individual groups based on the whole molecular trajectory instead of analysing individual time points.

**LMMS for differential expression analysis (LMMSDE)**: We extended the LMMS modelling framework to test between groups, across time, and for interactions between groups and time as follows. Suppose we have *R* different groups of subjects, with *h*_*i*_ denoting the group for each subject *i*. Further, we define *h*_*ir*_ to be the indicator for the *r* ^*th*^ group, that is, *h*_*ir*_ = 1 if *h*_*i*_ = *r* and 0 otherwise. Starting from the model in Eq 3 which is fit for a single group, we can extend our formulation to allow for variations to the mean curve depending on which group contains each subject. Thus the mean curve for each group *f*_*h*_*i*__ in the full LMMSDE model is given by:

$$\begin{array}{c} f_{h_{i}}\left(t_{ij}\right)=\beta_{0}+\beta_{1}t_{ij}+\sum_{k=1}^{K}u_{k}{\left(t_{ij}-\kappa_{k}\right)}_{+}\\ +\sum_{r=2}^{R}h_{ir}\left(\alpha_{0r}+\alpha_{1r}t_{ij}\right)+\sum_{r=2}^{R}h_{ir}\{\sum_{k=1}^{K}v_{rk}{\left(t_{ij}-\kappa_{k}\right)}_{+}\}. \end{array}$$ (6)

For each *r* = 1, …, *R*, *α*_**0**_ = *α*_0*r*_ are the differences in intercept between each group and the first group; *α*_**1**_ = *α*_1*r*_ are the differences in slope between each group and the first group; and *v*_*rk*_ are the differences in spline coefficients between each group and the first group.

We can test different hypotheses depending on which parameters are equal to zero. Firstly, for a single group, ∀*r* > 1, we have *h*_*ir*_ = 0, and time effects will be detected only if the goodness of fit of this model is better than the null model which fits only the intercept. Secondly, to detect differences between groups, we set *α*_**1**_ = 0 and *β*_1_ = 0, and test a goodness of fit against the null model which also has *h*_*ir*_ = 0. Finally, if we include all parameters we can model the group * time interactions, by allowing different slopes and intercepts in the different groups. We compare this to the null model where the effects over time do not differ between groups. For each case we compared the fit of the expanded model from Eq (6) with the corresponding null model using the likelihood ratio test as implemented in the anova function from the R package nlme [37].

**Comparisons with LIMMA**: We compared our approach to LIMMA [19], which is a set of methods for microarray data analysis integrating empirical Bayes approaches with linear models. We tested for differences over time and between groups using the following two-step process. First, linear spline models were fitted over time for every group. Second, contrasts of coefficients from the fits were tested for significance. Correction for multiple testing was applied for both methods for a significance level of 0.05 using the FDR approach from [6]. Note that no filtering was performed before differential expression analysis for two reasons: first, we wanted to compare the results based only on differences between models, and not on differences in filtering approaches; second, p-values derived from LIMMA are based on posterior estimates and the removal of non-informative profiles before analysis would therefore bias the results.

## Results

### Quality control and filtering

**Fig 4:**
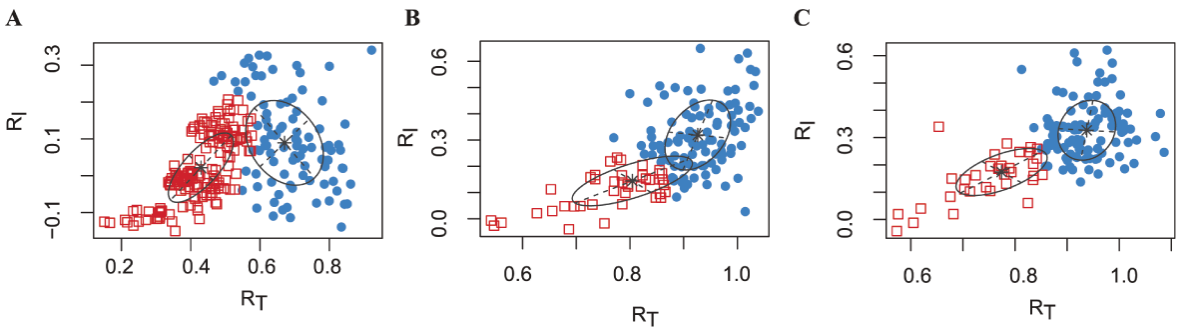
Clustering of filter ratios on proteomic datasets. Scatterplots of filter ratios *R*_*T*_ on the x-axis against *R*_*I*_ on the y-axis for **A**) iTraq breast cancer dataset and **B**) and **C**) the iTraq kidney rejection dataset for group Allograft Rejection (AR) and Non-Rejection (NR) respectively. Colors indicate clusters from a 2-cluster model-based clustering, with red squares indicating molecules that cluster as ‘informative’ and will remain in the analysis and blue circles indicating ‘non-informative’ molecules that will be removed prior to analysis.

We considered the performance of our filtering procedure in both proteomics and transcriptomics datasets. On the iTraq breast cancer (Fig 4A) and iTraq kidney rejection data (Fig 4B, 4C) we obtained one cluster with low *R*_*T*_ and *R*_*I*_ ratios, and a second cluster with high values for the two ratios. We therefore removed the molecules from that second cluster. Similar types of clusters were observed for all transcriptomics datasets.

**Fig 5:**
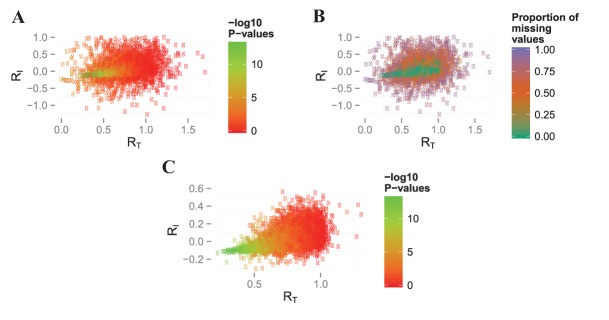
Filtering ratios of the *Mus musculus* data. The filter ratios *R*_*T*_ and *R*_*I*_ were calculated for every molecule. Colors in **A**) indicate the -log10(p-values) for differential expression over time and in **B**) the proportion of missing values. **C**) is after discarding profiles with > 50% of missing values, with colors as in **A**).

In total, between 35% and 76% of the data were removed (Table 1). As our filtering process is based on identifying high signal to noise ratios over time, we expected the remaining profiles to be enriched for those differentially expressed over time. In Fig 5 we present the relationship between the filter ratios and p-values obtained from performing a differential expression analysis over time using the new LMMSDE approach. We highlight the decrease in p-values when there is a decrease in filter ratios in the *M. musculus* data (similar results were obtained in the other datasets). However, contrary to our expectation we also observed for some low *R*_*T*_ values large p-values. We can explain the large p-values for low *R*_*T*_ in Fig 5A by the presence of a large number of missing values (> 50%) in the raw data (Fig 5B). Subsequently, the removal of profiles with more than 50% of missing data resulted in the expected previously described trend of decreasing p-values with decreasing *R*_*T*_ and *R*_*I*_ values (Fig 5C).

**Table 1:** Types of models used to summarize profiles. The number (proportion) of profiles modelled with each model selected by our proposed LMMS approach. Models are abbreviated as linear (LIN), spline (SPL), subject-specific intercept (SSI), and subject-specific intercept and slope (SSIS). Models were applied to cell line breast cancer data (Cell), *Saccharomyces paradoxus* evolution data (Yeast), *Mus musculus* chemotherapy data (Mouse), and *Homo Sapiens* kidney rejection Non-Rejection (NR) data (Human). The row ‘Removed’ indicates the percentage of filtered profiles using the 2-cluster model-based clustering on *R*_*T*_ and *R*_*I*_.

Model	Cell	Yeast	Mouse	Human
LIN Eq 1	93 (.55)	125 (.035)	205 (.1)	3 (.091)
SPL Eq 2	75 (.45)	3427 (.95)	1769 (0.87)	3 (.091)
SSI Eq 4		30 (.008)	56 (.028)	10 (.3)
SSIS Eq 5		2(.0005)	3 (.002)	17 (.51)
# Modelled	264	3586	2033	33
% Removed	36	35	67	76

### Modelling

The power of our LMMS modelling lies in its ability to adaptively fit the complexity of the data. Since some molecules are more prone to subject-specific variations than others, we generally expect that a single model will be insufficient to appropriately model all types of trajectories. We illustrate our point through the application of LMMS to datasets with increasing organism complexity, from cell lines measured in a controlled environment to *H. sapiens* with varying genetic and environmental factors. Table 1 shows that the proportion of complex models required to summarize molecule profiles increases with organism complexity.

### Clustering

**Fig 6:**
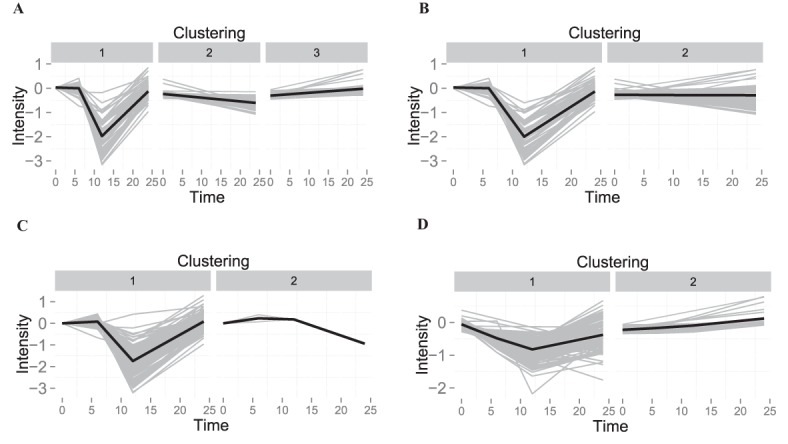
Clustering of the iTraq breast cancer dataset. Clustering was performed on the summarized profiles obtained from **A**) Linear Mixed Model Spline (LMMS), **B**) Derivative Linear Mixed Model Spline (DLMMS), **C**) mean and **D**) Smoothing Splines Mixed Effects (SME). The best clustering algorithm and the best number of clusters were chosen according to the Dunn index. In **A**), **B**) and **D**) we used hierarchical clustering and in **C**) Partitioning Around Medoids (PAM) clustering. The x-axis represents time (in hours) and the y-axis intensity in terms of *log*_2_ transformed protein abundance.

We compared clustering of profiles from the iTraq breast cancer dataset which had been modelled with mean, SME, LMMS and DLMMS (Fig 6). For each method, performance of different algorithms and optimal number of clusters (from two to nine) was assessed using the Dunn index.

This criterion resulted in different selections for these two quantities for the four modelling approaches (S3 File). This in turn led to different shapes of profiles being represented in the clusters selected for each modelling approach. The LMMS modelled profiles could be grouped into the largest number of clusters, which allowed better discrimination between temporary changes and linear increases/decreases over time when compared to the other approaches (Fig 6A).

We subsequently assessed the biological relevance of the proteins identified within each cluster with a GO term enrichment analysis. After removal of GO terms that contained only one molecule, we identified 62 unique enriched GO terms (adj. p-value ≤ 0.05, S1 Table) across all clusters and methods. Most of the methods identified some specific and unique GO terms: 9 for LMMS, 10 for mean and 1 for DLMMS (**Figure B** in S4 File). Clustering profiles using the methods mean and SME resulted in the enrichment of the intrinsic apoptotic signaling pathway (GO:0097193) which was found to be altered under IGF-1 stimulation [38]. Both LMMS and DLMMS found enriched biological function for programmed cell death (GO:0012501) and negative regulation of programmed cell death (GO:0060548), which were also shown to be altered under IGF-1 stimulation [39].

Interestingly, among the enriched GO terms identified by LMMS or DLMMS we observed biological processes involved in glucose metabolic processes (GO:0006006), glycolysis (GO:0006096) and gluconeogenesis (GO:0006094). These processes play an important role in cancer progression [40], indicating that growth of the cancer cells may be stimulated by IGF-1. The small cluster numbered 3 for the LMMS profiles was the only cluster in any of the methods that identified profiles with monotonically increasing expression. These profiles were involved in biological processes such as gluconeogenesis (GO:0006094), G-protein coupled receptor binding (GO:0001664) and phosphorylation (GO:0016310), which have all been shown to be an important part of the IGF-1 signalling cascade in association with cancer [41], [39, 42] (S2 Table).

## Differential Expression Analysis

### Simulated data

We compared the proposed LMMSDE with LIMMA on the unfiltered simulated data with varying expression patterns and levels of noise. For each scenario, we recorded how many of 50 differentially expressed molecules were detected as significant after correction for multiple testing and calculated average sensitivity and specificity over all 100 replicates (Table 2). Overall, LIMMA and LMMSDE performed very well at a noise level similar to what was observed in real data (Noise = 1), with both specificity and sensitivity higher than 0.96 for fold change (FC) levels above 1.5. For lower fold changes (FC = 1.25), differences between the two methods became more apparent. LMMSDE was still very sensitive (0.98) to group differences, but the performance of LIMMA (0.85) dropped. With increased variability, the difference between methods became more dramatic, as LMMSDE was much more sensitive compared to LIMMA except for the lowest FC. Similar trends were observed for all differential expression tests performed and LMMSDE consistently outperformed LIMMA for low FC and high variability.

**Table 2:** Simulation results. Averaged sensitivity for LMMSDE and LIMMA after 100 simulations. Differential expression between groups and/or time was tested with increasing noise and fold change (FC) levels.

Effect	Noise	FC	LMMSDE	LIMMA	Effect	Noise	FC	LMMSDE	LIMMA	Effect	Noise	FC	LMMSDE	LIMMA
		1.25	0.877	0.793			1.25	0.98	0.85			1.25	0.96	0.927
	1	1.5	0.981	0.963		1	1.5	0.997	0.976		1	1.5	0.993	0.987
time		2	0.997	0.992	group		2	0.999	0.995	group		2	0.999	0.998
		1.25	0.044	0.019			1.25	0.66	0.053	* time		1.25	0.347	0.124
	3	1.5	0.667	0.354		3	1.5	0.943	0.494		3	1.5	0.845	0.703
		2	0.939	0.838			2	0.986	0.881			2	0.965	0.938

### iTraq kidney rejection data

We performed a differential expression analysis on the iTraq kidney rejection dataset to illustrate our LMMSDE analysis on complex and real data. In addition to applying the differential expression approaches LIMMA and LMMSDE on the full data set as in the simulated case study, we also applied our filtering approach for multiple conditions and removed profiles that were identified as non-informative in both conditions (64% of profiles were removed) before LMMSDE analysis. Filtering before differential expression analysis was only applied for LMMSDE, since removal of non-informative profiles should increase statistical power without biasing results. In contrast, filtering before LIMMA analysis affects posterior estimates and can bias p-values.

We compared LMMSDE and LIMMA in terms of the number of proteins declared as differentially expressed between the two groups and investigated their biological relevance with respect to the biological questions from the study. Two analyses were performed: to identify the molecules with significant differences between groups, and to identify molecules showing significant group*time interactions leading to different trends between the two groups over time. While no differentially expressed molecules were identified by LIMMA for either group or interaction effects, LMMSDE identified 35 differentially expressed proteins with a group effect and 12 proteins with a significant interaction effect (FDR adjusted p-value < 0.05). On the filtered dataset LMMSDE identified 13 molecules with a significant group effect and nine molecules with a significant interaction effect. Note that these differentially expressed proteins were also identified in the analysis of the full dataset. The effect size of differential proteins identified with both group and interaction effects tended to be small, with a magnitude of average fold change of < 1.5.

For the 13 (three not annotated) molecules that were declared as differentially expressed between groups, the top enriched biological process (Table 3) was the negative regulation of endopeptidase activity (GO:0010951). This is of interest since an increase in the activity of serum neutral endopeptidase has been shown to play an important role in acute renal graft rejection [43]. An additional enrichment observed in the complement activation (GO:0006956) is also of biological relevance as innate immune responses are major causes of graft rejection [44]. Therefore, these molecules present good candidate biomarkers for the prediction of allograft rejection. Some differentially expressed molecules were also present in biological processes involving platelet degranulation (GO:0002576) and platelet activation (GO:0030168), which have been shown to contribute to hyperacute rejection of both allografts and xenografts [45].

**Table 3:** iTraq kidney rejection dataset: Gene Ontology (GO) term enrichment analysis. GO term enrichement analysis based on the proteins identified by LMMSDE as differentially expressed between Allograft Rejection (AR) and Non-Rejection (NR) patients after filtering using a 2-cluster model-based clustering based on *R*_*T*_ and *R*_*I*_. The top GO biological processes are listed along with their FDR adjusted p-value and log odds ratio (OR).

GO	GO Description	adj. p-value	log(OR)
GO:0010951	negative regulation of endopeptidase activity	4.30e-03	5.57
GO:0006956	complement activation	6.40e-03	6.45
GO:0006958	complement activation, classical pathway	6.40e-03	6.36
GO:0002576	platelet degranulation	8.30e-03	5.67
GO:0045471	response to ethanol	8.30e-03	5.55
GO:0042593	glucose homeostasis	8.80e-03	5.43
GO:0006935	chemotaxis	1.00e-02	5.14
GO:0007596	blood coagulation	1.00e-02	3.84
GO:0030168	platelet activation	1.70e-02	4.30

Out of the nine molecules (1 not annotated) with a significant interaction between group and time, the most promising protein differentially expressed was IQ calmodulin-binding motif-containing protein 1 (IQCB1). This protein is particularly relevant to this study, as it is a nephrocystin protein localized to the primary cilia of renal epithelial cells. Mutations in this gene were shown to be strongly associated with Senior-Løken-Syndrome Type 5, a disorder causing nephronophthisis and renal failure [46].

## Discussion

Thus far, very few methods have been developed to analyse high-throughput time course ‘omics’ data. Statistical analysis is challenging due to the high level of noise relative to signal in such data, and the time measurements add an extra dimension of variability both within and among subjects. Our data-driven approach focuses on magnifying the inherent signal, by removing non-informative profiles that potentially interfere in downstream analysis, and by using a linear mixed model spline framework to account for subject-specific variability. This procedure provides clearer signals in both clustering and differential expression analysis.

The filtering of non-informative profiles is an important first step in analysis, as such profiles otherwise introduce noise and reduce statistical power in downstream clustering and differential expression steps [47, 26, 48]. We have extended the standard deviation filter for static microarray experiments proposed by [26] and introduced a computationally fast approach accompanied by useful visualizations (see Figs 4, 5). We demonstrated that our filtering approach was effective at discriminating informative from non-informative profiles by comparing the values of our filter statistics *R*_*T*_ and *R*_*I*_ with the test statistics from differential expression analysis over time.

For multiple treatment groups, we filtered separately for each group, removing only molecules identified as non-informative in both groups. An alternative option would be to calculate the ratios for each group separately, but apply the model-based clustering on all ratios from all groups. We found very little differences compared to a filtering approach applied on each treatment group. Using one of these approaches, it is possible that molecules that vary between groups, but show little change over time could be removed. However, these molecules, though differentially expressed, would be detected in a cross-sectional study, and are most likely not of primary interest in time course studies where the focus is on molecules changing expression over time.

In spite of the clear relationship between differential expression and filter ratios, we found the selection of thresholds to be challenging. Threshold choice can be affected by a variety of issues such as level of missing data and the number of replicates at each time point. In our analysis, we applied 2-cluster model-based clustering on the ratios to discriminate informative from non-informative profiles. However, we suggested guidelines to address these issues and our R package lmms allows the user to set their own thresholds. A drawback of our proposed filtering method is the requirement of the same sampled time points across subjects, and the need for at least three replicates per time point. If these do not hold, it may be necessary to collapse time points into bins prior to analysis to have sufficient density of data. Further investigation of filters allowing for less constrained sampling could be very useful for adaptive sampling designs.

Current modelling approaches for time course data fit the same statistical model to each molecule, allowing for either subject-specific intercepts [21] or subject-specific intercepts and slopes [22]. However, we expect that effects of environmental and/or genetic factors on expression vary for individual molecules. Therefore, as acknowledged by [22], the use of only one model for all molecules has the serious limitation of under-smoothing or over-smoothing the representative profiles. Our method improved upon existing methods by allowing the data to drive the complexity of the models rather than having a single fixed model. Our analyses showed that model flexibility was necessary, and that not only the choice of model was molecule-dependent, but also that the proportion of complex models increased with organism complexity. Our LMMS modelling approach was applied to a range of typical time course transcriptomics and proteomics experiments with different numbers of replicated measurements (3 to 20 per time point) and time points (4 to 6), and we expect the approach to be scalable and highly valuable for larger experiments.

In this study we clustered time course data based on their summarized profiles to identify groups of molecules representing relevant molecular processes. We did not consider here clustering of subjects to identify groups with similar sub-phenotypes. However, similar approaches can be applied to this alternate biologically interesting question [49].

Clustering analysis relies not only the choice of algorithm, but also on the number of clusters and the distance metric. There are a variety of options available for all of these, but we have focused on common choices in this study, and expect that other options would produce similar results. We observed that application of different modelling approaches (e.g. mean, SME, LMMS) resulted in different input data structure to the clustering algorithms. As clustering outputs are highly dependent on the input data structure [50], it was not surprising that the clustered patterns and the optimal number of clusters varied across algorithms and consequently led to differences in biological interpretation between clusters. We showed that applying LMMS prior to clustering allowed the identification of a cluster of biologically interesting co-expressed genes. This highlights the importance of accurately modelling before clustering.

Differential expression analysis is often based on an underlying model of the data which attempts to explain changes over time, between group, and through interactions while simultaneously accounting for noise in the data. We compared an approach based on linear models, LIMMA, with our approach, LMMSDE, which is based on our linear mixed model spline framework. An alternate spline-based approach is EDGE [21], but a comparative analysis was not feasible with the current version of their package. In our simulation study, we showed that LMMSDE gave superior results as it led to higher sensitivity, particularly for small fold changes and high noise levels. Consequently, in a real biological data setting, LMMSDE identified highly relevant differentially expressed molecules while LIMMA identified none. We note that for both LMMSDE and LIMMA, the choice of spline basis can have a major effect on differential expression analysis. We have focused here only on the linear penalized spline basis, but there are many alternatives available, including the cubic spline and penalized cubic spline, which we have implemented as options in our R package lmms. Higher-degree polynomials may provide additional power for detection of differential expression over time when the profiles display nonlinear behaviour, as in cluster 1 (Fig 6) for the breast cancer data.

An additional benefit of LMMSDE was the ability to first perform filtering, which reduced the number of tests performed and increased our ability to detect truly differentially expressed molecules. The same type of analysis could not be performed with LIMMA, as its test statistic is based on an empirical Bayes approach using posterior estimators for degree of freedom and standard deviation. Therefore, a filtering of low variance molecules would affect posterior estimates [26] and bias the results. By proposing a unified framework we thus achieve gains throughout the entire statistical analysis process.

## Conclusion

We proposed a novel framework for analysing time course ‘omics’ data, unifying quality control and filtering, modelling, and analysis in a linear mixed model spline framework. The first step ensures the reproducibility and interpretability of the data. The second step is a highly flexible data-driven approach aimed at modelling high-throughput data with potentially different noise levels and trajectories over time. It can handle missing values, has low computational burden, and avoids arbitrary input parameters. In the third step, similarities between profiles can be assessed through clustering, or differences over time and between groups can be assessed through LMMSDE. The unification of our modelling with clustering led to the identification of biologically relevant profile clusters. The unification of our modelling with differential expression analysis outperformed LIMMA in the situations of high noise levels and low fold changes. In application of LMMSDE to real data, this higher sensitivity resulted in novel identification of differentially expressed molecules biologically relevant to kidney rejection. The LMMS framework is implemented in the R package lmms and is freely available for download from CRAN.

## Supporting Information

S1 File Example of simulated profiles with a time effect (**Figure A**), a group effect (**Figure B**) and a group and time interaction (**Figure C**). The noise level is equal to that in the kidney rejection data and the groups of each individual are indicated in grey full lines (group 1) or black dashed lines (group 2). In **Figure A** the expression increases over time with a fold change of *log*(2) from the first to the last time points, in **Figure B** the fold change between the two groups is equal to *log*(2), in **Figure C** the profiles measured on individuals from group 1 (group 2) increase (decrease) over time with a fold change of *log*(2).
(PDF)

S2 File Relationship between filter ratios, differential expression and presence of missing values in multiple datasets. Filter ratios *R*_*T*_ (x-axis) and *R*_*I*_ (y-axis) are shown for: simulated data (**Figure A**); iTraq breast cancer data (**Figure B**); *Saccharomyces paradoxus* evolution data (**Figure C**); iTraq kidney rejection Allograft Rejection (AR) data (**Figure D**). Molecules are coloured according to −*log*_10_ p-values for Linear Mixed Model Spline for Differential Expression analysis (LMMSDE) test for differential expression over time (first column) and the proportion of missing values (second column).
(PDF)

S3 File Internal stability of iTraq breast cancer clusters: using the mean (**Figure A**); Smoothing Splines Mixed Effects (SME) (**Figure B**); Linear Mixed Model Spline (LMMS) (**Figure C**) and Derivative LMMS (DLMMS) (**Figure D**) for summarizing the profiles across the biological replicates. Dunn indices are displayed for a number of clusters varying from two to nine with the five different cluster algorithms: hierarchical clustering (HC), kmeans (KM), Partitioning Around Medoids (PAM), model-based (model) and Self-Organizing Maps (SOM). Higher Dunn indices indicate better clustering performance.
(PDF)

S4 File iTraq breast cancer cluster Gene Ontology (GO) term enrichment analysis. Venn diagram of significantly enriched GO terms identified by clustering of the mean, Smoothing Splines Mixed Effects (SME), Linear Mixed Model Spline (LMMS) and Derivative LMMS (DLMMS) before (**Figure A**) and after (**Figure B**) removing GO terms that contained only one molecule.
(PDF)

S1 Table iTraq breast cancer dataset: overlapping enriched Gene Ontology (GO) terms. Shown are the GO terms identified concordantly by clustering of at least two of the modelling approaches (Linear Mixed Model Spline (LMMS), Derivative LMMS (DLMMS), mean or Smoothing Splines Mixed Effects (SME)).
(PDF)

S2 Table iTraq breast cancer dataset: unique identified Gene Ontology (GO) terms. Enriched GO terms uniquely identified by clustering of the profiles modelled by the different approaches considered. For each enriched term, the cluster number (Cluster), the number of molecules with GO terms in that cluster (Counts), the number of molecules in the data with that GO term (NMol), the number of molecules in the cluster (Size), the GO description, ontology (Ont), false discovery rate adjusted p-value (adj. p), and log odds ratio (OR) are given. The table is sorted by p-value within each cluster. Linear Mixed Model Spline (LMMS); Derivative LMMS (DLMMS) and Splines Mixed Effects (SME) use hierarchical clustering while the mean uses PAM clustering. For LMMS three clusters were identified, while two clusters were identified for DLMMS, mean and SME.
(PDF)
