# Supplementary material for: A Linear Mixed Model Spline Framework for Analysing Time Course ‘Omics’ Data
Source: PLoS One. 2015 Aug 27;10(8):e0134540. doi: 10.1371/journal.pone.0134540 (PMC4551847; doi:10.1371/journal.pone.0134540)

S1 Generation of simulated data

We simulated 100 datasets with different case scenarios including three different fold changes (FC), three types of differential expression (DE) profiles and two different noise levels. Each simulated dataset consisted of 140 profiles measured on six time points and 40 individuals. Using the NR patients from the iTraq kidney rejection dataset, we first extracted the individual variance σ_U^2 and error variance σ_ϵ^2 from the individual-specific random intercepts and slopes from our fourth LMMS model (Eq. 5 in the main paper). From these estimates, we generated DE profiles as described below.

Let y_{ij} be the simulated expression of a molecule for individual (or biological replicate) i where $i = 1, 2, ..., n$, $j = 1, 2, ..., m_i$ with $n = 40$ is the sample size and $m_i = 6$ is the the number of time points observed for each individual i. Starting from flat (null) profiles, we added a randomly assigned individual effect called subject.effect$_{ij} \sim \mathcal{N}(0, \sigma_U^2)$ as well as a molecule-specific error, $\epsilon_{ij} \sim \mathcal{N}(0, \sigma_\epsilon^2)$. We then randomly assigned to 50 molecules different types of differential expression depending on the fold change levels (referred to as de.effect$_j$) and noise levels ϵ_{ij}. The remaining 90 molecules were modelled with no differential expression effect *(i.e.* de.effect$_j = 0$).

Each simulated expression trajectory y_{ij} can be written as:

$$y_{ij} \sim 0 + \text{subject.effect}_{ij} + \text{de.effect}_j + \epsilon_{ij},$$

with

- subject.effect$_{ij} \sim \mathcal{N}(0, \sigma_U^2)$ is the stochastic subject-specific noise,
- de.effect$_j = 0$ if the the molecule is not differentially expressed between the two groups otherwise it can take different values depending on the intended test:
  - **Time effect**: de.effect$_j$ takes values between $log(1)$ and $log(FC)$ depending on the time point with FC=1.25, 1.5 or 2,
  - **Group effect**: de.effect$_j$ takes the value $log(FC)$ with FC=1.25, 1.5 or 2,
  - **Group*Time effect**: de.effect$_j$ takes values between $log(1)$ and $log(FC)$ depending on the time point, the fold change FC=1.25, 1.5 or 2 and the group to which the individual belongs.
- $\epsilon_{ij} \sim \mathcal{N}(0, \sigma_\epsilon^2)$ is the stochastic noise and can take the following values:
  - **Noise 1**: $\epsilon_{ij} \sim \mathcal{N}(0, \sigma_\epsilon^2)$
  - **Noise 3**: $\epsilon_{ij} \sim \mathcal{N}(0, 3 * \sigma_\epsilon^2)$.

S1 File illustrates some simulated profiles for the time, group and group * time interaction cases.

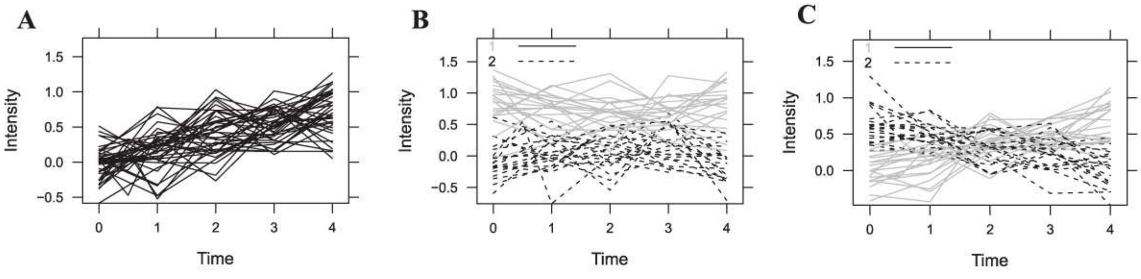

Supplement: S1 File — The noise level is equal to that in the kidney rejection data and the groups of each individual are indicated in grey full lines (group 1) or black dashed lines (group 2). In Figure A the expression increases over time with a fold change of log(2) from the first to the last time points, in Figure B the fold change between the two groups is equal to log(2), in Figure C the profiles measured on individuals from group 1 (group 2) increase (decrease) over time with a fold change of log(2). (PDF) [file pone.0134540.s001.pdf]
