# Supplementary material for: A Linear Mixed Model Spline Framework for Analysing Time Course ‘Omics’ Data
Source: PLoS One. 2015 Aug 27;10(8):e0134540. doi: 10.1371/journal.pone.0134540 (PMC4551847; doi:10.1371/journal.pone.0134540)
Supplement: S2 File — Filter ratios R T (x-axis) and R I (y-axis) are shown for: simulated data (Figure A); iTraq breast cancer data (Figure B); Saccharomyces paradoxus evolution data (Figure C); iTraq kidney rejection Allograft Rejection (AR) data (Figure D). Molecules are coloured according to −log 10 p-values for Linear Mixed Model Spline for Differential Expression analysis (LMMSDE) test for differential expression over time (first column) and the proportion of missing values (second column). (PDF) [file pone.0134540.s002.pdf]

S2 Quality control and filtering

High quality input data is crucial for reproducibility and interpretability, particularly in time course ‘omics’ where there are a large number of molecules, often observed across relatively few individuals and time points.

Motivation for our definition of filter ratios. In the main paper, we have defined s_T as the average standard deviation (SD) per time point, s_I the average SD per individual and s_M as the SD for the molecule over all individuals and time points. We introduced the definitions of the two filter ratios, $R_T = \frac{s_T}{s_M}$ and $R_I = 1 - \frac{s_I}{s_M}$ to quickly assess the profile quality and filter out ‘noisy’ profiles prior to analysis. The accompanying R package `lmms` provides an example, and gives the user the flexibility of choosing the R_T and R_I threshold values.

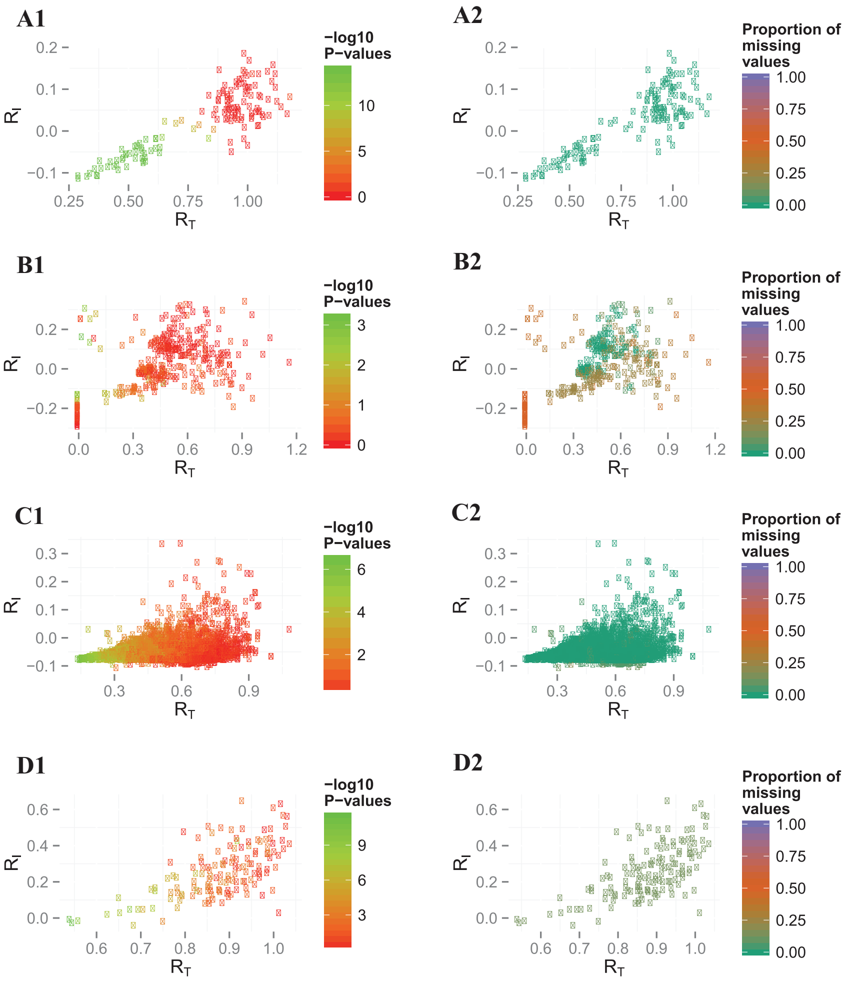

Relationship between filter ratios, differential expression and presence of missing values. To characterise the behaviour of the filter ratios on different datasets, we consider their relationship with differential expression over time (using our approach LMMSDE) and proportion of missing data. Our first observation is that lower filter ratios typically correspond to more significantly expressed molecules, so our choice of thresholds is unlikely to remove molecules differentially expressed over time (column 1 in S2 File). Our second observation is that molecules with large amounts of missing data may not be excluded using the filtering thresholds (column 2 in S2 File and especially case study Figure **B2** in S2 File). We therefore decided to remove profiles with more than 50% missing values.
