# Supplementary material for: A Linear Mixed Model Spline Framework for Analysing Time Course ‘Omics’ Data
Source: PLoS One. 2015 Aug 27;10(8):e0134540. doi: 10.1371/journal.pone.0134540 (PMC4551847; doi:10.1371/journal.pone.0134540)
Supplement: S3 File — using the mean (Figure A); Smoothing Splines Mixed Effects (SME) (Figure B); Linear Mixed Model Spline (LMMS) (Figure C) and Derivative LMMS (DLMMS) (Figure D) for summarizing the profiles across the biological replicates. Dunn indices are displayed for a number of clusters varying from two to nine with the five different cluster algorithms: hierarchical clustering (HC), kmeans (KM), Partitioning Around Medoids (PAM), model-based (model) and Self-Organizing Maps (SOM). Higher Dunn indices indicate better clustering performance. (PDF) [file pone.0134540.s003.pdf]

S3 Clustering

Selection of cluster algorithm. We have compared several clustering approaches: one model-based algorithm (R package `mclust`; Fraley and Raftery, 2002) and four algorithms based on a distance metric: Hierarchical Clustering (HC), kmeans (KM), Partitioning Around Medoids (PAM, R package `cluster`; Maechler *et al.*, 2013), and Self-Organizing Maps (SOM, R package `kohonen`; Wehrens and Buydens, 2007). We assessed the performance of the five clustering algorithms with an internal consistency criterion, the Dunn index, while varying the number of clusters from two to nine (S3 File).

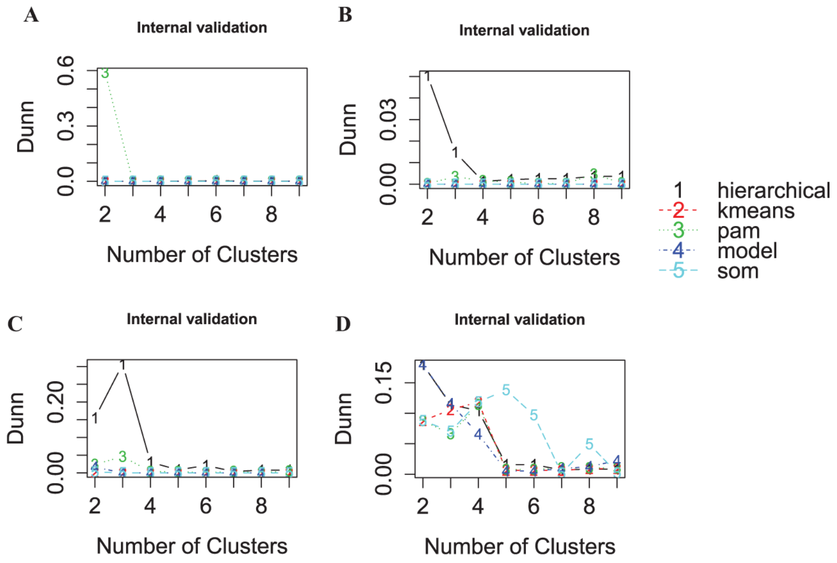

References

Fraley, C. and Raftery, A. (2002). Model-Based Clustering, Discriminant Analysis, and Density Estimation. *J. Amer. Statist. Assoc.*, **97**(458), 611–631.

Maechler, M., *et al.* (2013). *cluster: Cluster Analysis Basics and Extensions*. R package version 1.14.4.

Wehrens, R. and Buydens, L. (2007). *Self- and Super-organising Maps in R: the kohonen package*. R package version 2.0.14.
