# Supplementary material for: A Linear Mixed Model Spline Framework for Analysing Time Course ‘Omics’ Data
Source: PLoS One. 2015 Aug 27;10(8):e0134540. doi: 10.1371/journal.pone.0134540 (PMC4551847; doi:10.1371/journal.pone.0134540)

S4 Cluster GO enrichment analysis

GO term enrichment analysis as described in the main manuscript was used to gain additional insight into the biological relationships between molecule profiles belonging to the same cluster. We calculated p-values using the hypergeometric distribution based on the number of molecules in the domain of interest. S4 File shows the overlap of enriched GO terms identified by clustering the different modelling approaches on the iTraq breast cancer dataset (mean, SME, LMMS and DLMMS) before and after removing GO terms that contained only one molecule. Table S1 lists the common enriched GO terms in at least two of the mean, SME and LMMS and DLMMS clusters, while Table S2 lists the enriched GO terms uniquely identified by each set of clusters.

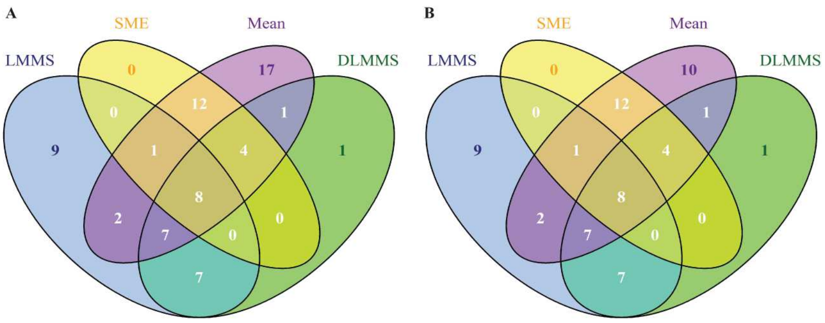

Supplement: S4 File — Venn diagram of significantly enriched GO terms identified by clustering of the mean, Smoothing Splines Mixed Effects (SME), Linear Mixed Model Spline (LMMS) and Derivative LMMS (DLMMS) before (Figure A) and after (Figure B) removing GO terms that contained only one molecule. (PDF) [file pone.0134540.s004.pdf]
