# Supplementary material for: A Linear Mixed Model Spline Framework for Analysing Time Course ‘Omics’ Data
Source: PLoS One. 2015 Aug 27;10(8):e0134540. doi: 10.1371/journal.pone.0134540 (PMC4551847; doi:10.1371/journal.pone.0134540)
Supplement: S1 Table — Shown are the GO terms identified concordantly by clustering of at least two of the modelling approaches (Linear Mixed Model Spline (LMMS), Derivative LMMS (DLMMS), mean or Smoothing Splines Mixed Effects (SME)). (PDF) [file pone.0134540.s005.pdf]

GO	GO Description	Method
GO:0045095	keratin filament	LMMS, DLMMS, Mean, SME
GO:0005882	intermediate filament	LMMS, DLMMS, Mean, SME
GO:0044822	poly(A) RNA binding	LMMS, DLMMS, Mean, SME
GO:0005634	nucleus	LMMS, DLMMS, Mean, SME
GO:1900740	positive regulation of protein insertion into mitochondrial membrane involved in apoptotic signaling pathway	LMMS, DLMMS, Mean, SME
GO:0005200	structural constituent of cytoskeleton	LMMS, DLMMS, Mean, SME
GO:0005198	structural molecule activity	LMMS, DLMMS, Mean, SME
GO:0005719	nuclear euchromatin	DLMMS, Mean, SME
GO:0005615	extracellular space	DLMMS, Mean, SME
GO:0005515	protein binding	DLMMS, Mean, SME
GO:0005737	cytoplasm	DLMMS, Mean, SME
GO:0005829	cytosol	LMMS, Mean, SME
GO:0031982	vesicle	LMMS, DLMMS, Mean
GO:0030529	ribonucleoprotein complex	LMMS, DLMMS, Mean
GO:0006096	glycolytic process	LMMS, DLMMS, Mean
GO:0031012	extracellular matrix	LMMS, DLMMS, Mean
GO:0004743	pyruvate kinase activity	LMMS, DLMMS, Mean
GO:0023026	MHC class II protein complex binding	LMMS, DLMMS, Mean
GO:0003725	double-stranded RNA binding	LMMS, DLMMS, Mean
GO:0097193	intrinsic apoptotic signaling pathway	Mean, SME
GO:0001895	retina homeostasis	Mean, SME
GO:0031490	chromatin DNA binding	Mean, SME
GO:0021762	substantia nigra development	Mean, SME
GO:0097110	scaffold protein binding	Mean, SME
GO:0005925	focal adhesion	Mean, SME
GO:0070062	extracellular vesicular exosome	Mean, SME
GO:0019904	protein domain specific binding	Mean, SME
GO:0072562	blood microparticle	Mean, SME
GO:0004601	peroxidase activity	Mean, SME
GO:0006605	protein targeting	Mean, SME
GO:0014069	postsynaptic density	Mean, SME
GO:0071901	negative regulation of protein serine/threonine kinase activity	Mean, SME
GO:0042470	melanosome	DLMMS, Mean
GO:0031093	platelet alpha granule lumen	LMMS, Mean
GO:0008285	negative regulation of cell proliferation	LMMS, Mean
GO:0000790	nuclear chromatin	LMMS, Mean
GO:0008544	epidermis development	LMMS, DLMMS
GO:0006006	glucose metabolic process	LMMS, DLMMS
GO:0005929	cilium	LMMS, DLMMS
GO:0030955	potassium ion binding	LMMS, DLMMS
GO:0012501	programmed cell death	LMMS, DLMMS
GO:0060548	negative regulation of cell death	LMMS, DLMMS
GO:0005975	carbohydrate metabolic process	LMMS, DLMMS
