# Supplementary material for: A Linear Mixed Model Spline Framework for Analysing Time Course ‘Omics’ Data
Source: PLoS One. 2015 Aug 27;10(8):e0134540. doi: 10.1371/journal.pone.0134540 (PMC4551847; doi:10.1371/journal.pone.0134540)
Supplement: S2 Table — Enriched GO terms uniquely identified by clustering of the profiles modelled by the different approaches considered. For each enriched term, the cluster number (Cluster), the number of molecules with GO terms in that cluster (Counts), the number of molecules in the data with that GO term (NMol), the number of molecules in the cluster (Size), the GO description, ontology (Ont), false discovery rate adjusted p-value (adj. p), and log odds ratio (OR) are given. The table is sorted by p-value within each cluster. Linear Mixed Model Spline (LMMS); Derivative LMMS (DLMMS) and Splines Mixed Effects (SME) use hierarchical clustering while the mean uses PAM clustering. For LMMS three clusters were identified, while two clusters were identified for DLMMS, mean and SME. (PDF) [file pone.0134540.s006.pdf]

Method	Cluster	Counts	NMol	Size	GO	GO Description	Ont	adj. p	log(OR)
LMMS	3	7	19	51	GO:0005681	spliceosomal complex	CC	1.50e-03	3.55
	3	6	16	51	GO:0006094	gluconeogenesis	BP	4.20e-03	3.56
	3	6	19	51	GO:0000287	magnesium ion binding	MF	9.10e-03	3.18
	3	3	4	51	GO:0001664	G-protein coupled receptor binding	MF	1.50e-02	5.79
	3	3	4	51	GO:0016234	inclusion body	CC	1.50e-02	5.79
	3	5	15	51	GO:0016310	phosphorylation	BP	1.80e-02	3.26
	3	3	5	51	GO:0031072	heat shock protein binding	MF	3.00e-02	4.79
	3	3	5	51	GO:0031397	negative regulation of protein ubiquitination	BP	3.00e-02	4.79
	3	3	5	51	GO:0042623	ATPase activity, coupled	MF	3.00e-02	4.79
DLMMS	2	3	3	130	GO:0001890	placenta development	BP	0.047	Inf
mean	2	2	11	2	GO:0008285	negative regulation of cell proliferation	BP	3.00e-03	Inf
	1	49	195	166	GO:0016020	membrane	CC	6.20e-03	1.11
	1	31	109	166	GO:0005886	plasma membrane	CC	1.10e-02	1.27
	1	13	33	166	GO:0005856	cytoskeleton	CC	2.20e-02	1.90
	1	8	16	166	GO:0030018	Z disc	CC	3.70e-02	2.49
	1	12	26	166	GO:0019901	protein kinase binding	MF	9.80e-03	2.30
	1	6	9	166	GO:0008092	cytoskeletal protein binding	MF	2.20e-02	3.48
	1	6	9	166	GO:0044325	ion channel binding	MF	2.20e-02	3.48
	1	9	20	166	GO:0003674	molecular function	MF	4.40e-02	2.21
	1	13	36	166	GO:0019899	enzyme binding	MF	4.80e-02	1.69
